# Psychological Capital Differs Among Rural Left-Behind Children and Is Associated With Emotional and Behavioral Problems

**DOI:** 10.3389/fpsyg.2021.565385

**Published:** 2021-07-05

**Authors:** Cuiying Zhu, Xiaolan Yin, Zhihua Li, Lujun Zhou

**Affiliations:** ^1^College of Public Administration and Law, Hunan Agricultural University, Changsha, China; ^2^College of Education, Hunan Agricultural University, Changsha, China; ^3^Guangxi Sub-Center of China Financial Literacy Education Synergy Innovation Center, Guangxi University of Finance and Economics, Nanning, China; ^4^School of Information and Statistics, Guangxi University of Finance and Economics, Nanning, China

**Keywords:** rural left-behind children, psychological capital, latent profile analysis, emotional and behavioral problems, prosocial behavior

## Abstract

This study aims to explore, through latent profile analysis (LPA), rural left-behind children’s psychological capital and its relationship with emotional and behavioral indicators. In this study, 677 rural-based left-behind children (average age 11.7 ± 1.58 years) in Hunan Province, China, were recruited and assessed using the Rural Left-behind Children’s Psychological Capital Questionnaire and the Children’s Strengths and Difficulties Questionnaire. We found that psychological capital was divided into three latent profiles: high (43.3% of the sample), medium (46.1%), and low (10.6%). Compared to the other two types, the children with low psychological capital returned higher scores for emotional symptoms, conduct disorder, hyperactivity and impulsivity, and peer-interaction problems, but lower prosocial behavior scores. Meanwhile, examination of the effects of gender and grade found that most of the elementary school students had high psychological capital, and that there was no significant difference among the groups in regard to gender. In summary, distinct differences in psychological capital were found among left-behind children, and the latent profiles were determined to be related to grade, emotional symptoms, hyperactivity and impulsivity, and prosocial behavior. There was also a significant difference in emotional and behavioral indicators across the different latent profiles.

## Introduction

China’s rapid development has resulted in many rural unskilled laborers moving to cities to seek employment, causing growth in the country’s migrant population (Wang et al., 2015). China’s urban-rural dual system and the existence of unequal access to social resources such as education and health care have caused most of the migrant workers to travel without their children; these children, who remain in their home regions, are often referred to as “left-behind children.” Specifically, scholars define left-behind children as children under 16 years of age who have not lived with one or both parents for 6 months or more (Wang and Mesman, 2015). In August 2018, there were 6.97 million left-behind children in China (Xinhuanet, 2018). One study found that the mental health of left-behind children is easily affected by negative environmental, personality, and social factors (Graham et al., 2012). Further, some of these children have serious mental health problems (Fellmeth et al., 2018), emotional and behavioral problems (such as depression; He et al., 2012), and anxiety (Tomşa and Jenaro, 2015). Living away from parents and having little connection with them may be an important environmental factor that causes emotional and behavioral problems among left-behind children (Johnson and Mcneil, 1998); thus, these left-behind children require more effective psychological resources to adapt. In addition to external factors, such as social support and recognition by others, psychological capital, which is an internal factor of psychological resources, leads to more lasting motivation toward individual development. In particular, developing psychological capital, which features the four factors of hope, efficacy, resilience, optimism (or “HERO”) is important for left-behind children (Youssef and Luthans, 2007). Psychological capital was originally defined by Luthans et al. (2007) as a core positive psychological element that can be effectively developed and managed and that can have a significant impact on the health and growth of individuals. Early studies have found that psychological capital can reduce unfavorable adaptation in left-behind children brought about by family disadvantages and effectively buffer the negative impact of life pressure that increases the sense of loneliness and decreases well-being in left-behind children. It can be seen that left-behind children with high levels of psychological capital have more positive cognitive thinking and better adaptability to life, better able to regulate negative emotions, and thus feel more happiness. In particular, according to the Psychological Capital Intervention, resilience activates an individual’s cognitive, emotional, and behavioral processes, and can alter their perception of the impacts of their external conditions (Russo and Stoykova, 2015). Recently, studies have suggested that the Psychological Capital Intervention could be used to develop interventions for left-behind college students that can help them enhance their ability and adaptability (Liang et al., 2018). However, few studies have been conducted on the psychological capital of school-age left-behind children. In recent years, Fan et al. (2015) applied the Psychological Capital Intervention to left-behind children in China, finding that positive psychological ability can improve children’s psychological and behavioral adaptation. In addition, to account for cultural differences, they developed a Chinese version of the psychological capital questionnaire for left-behind children (the Rural Left-behind Children’s Psychological Capital Questionnaire), and this comprises the five factors of self-reliance and perseverance, sensibility and appreciation, optimism and cheerfulness, tolerance and kindness, and self-confidence and aggressiveness. It closely resembles the HERO model developed by Luthans, and it is effective for measuring individual psychological and behavioral adaptation (Fan et al., 2015, 2018). The findings mentioned above are of great significance to the study of left-behind children’s psychological capital.

Notably, despite experiences of adverse effects as a result of exposure to bad environments, some left-behind children show good psychological development; a possible reason for this is that psychological capital is also affected by experiences. Early experiences of being left at home may promote the development of psychological capital, enabling children to develop positive qualities such as independence and resilience (Wen, 2011; Cao, 2017). Most previous studies have argued that left-behind children report lower levels of psychological capital compared to other children; however, other studies found no significant difference in psychological capital between left-behind children and former and non-left-behind children (Fan et al., 2017). This indicates that the psychological capital of left-behind children is relatively distinct; therefore, we assume that the psychological capital of left-behind children is heterogeneous.

This theory is supported by the fact that there are contrasting findings across existing studies, with some studies reporting that left-behind children have lower levels of psychological capital than other children, while other studies have reported that there is no significant difference between the psychological capital of left-behind children and that of normal children (Fan et al., 2017). Therefore, it can be concluded that there may be intergroup differences among left-behind children in terms of level of psychological capital.

Latent profile analysis (LPA) represents a good method of investigating this. LPA is an individual-centered research method that can help provide an additional scientific method of classifying left-behind children’s psychological capital, thereby helping researchers obtain a better understanding of the differences among individuals. LPA can also be called potential class analysis, because the explicit variable is a continuous variable rather than a class variable. LPA is also an individual-based analysis, so it can find the cause of the problem. In recent years, LPA has been widely applied in fields such as psychology (Hagan et al., 2016). Further, previous research involving this approach has confirmed that left-behind children in China are a differentiated group with differences in terms of psychological traits and other areas (Fan et al., 2017); however, there is, as yet, no conclusion regarding the classification of such children’s psychological capital.

In summary, this study investigates differences in left-behind children’s psychological capital, which can help such children make the best use of their qualities and help them prevent and resolve their emotional and behavioral problems. In particular, this paper explores the different subtypes of left-behind children’s psychological capital through LPA, compares the emotions and behaviors of left-behind children with differing psychological capital, and discusses the influence of sociodemographic factors on the classification of psychological capital. This may be the first study to explore the latent classes of psychological capital among left-behind children.

## Subjects and Methods

### Subjects

This study adopted a stratified random sampling method. At the end of November 2017, 5 counties were randomly selected from the more developed, generally developed, and poor counties in Hunan Province. Each county selected 2–3 primary and junior high schools. A total of 140 questionnaires were randomly selected from each grade in grades 4 to 6 and 7 to 8 in junior high school. A total of 700 questionnaires were distributed, and 677 valid questionnaires were finally recovered, with an effective recovery rate of 96.71%. These 677 students were in 4th–8th grade, and comprised 321 boys (47.4%) and 356 girls (52.6%); 363 were elementary school students (53.6%) and 314 were junior middle school students (46.4%), with an average age of 11.7 ± 1.58 years. All subjects were children who lived in rural areas and who had at least one parent working in a city for more than half a year.

### Measures

#### Rural Left-Behind Children’s Psychological Capital Questionnaire

To measure the children’s psychological capital, the Rural Left-behind Children’s Psychological Capital Questionnaire (Fan et al., 2015) was used. This questionnaire is specially designed to assess the psychological capital of left-behind children. The questionnaire comprises 25 items and five factors: self-reliance and perseverance, sensibility and appreciation, optimism and cheerfulness, tolerance and kindness, and self-confidence and aggressiveness. Each child receives a score between 1 (if the child possesses the five factors) and 5 (if the child does not possess any of the five factors). Example items include “I am good at resisting all kinds of temptations” and “I often want to repay my parents and other elders in the future.” Except for Item 19, “I don’t see hope for my own future,” which is a positive score, the scores of other positively expression items are reverse-scored. Meanwhile, the sum of the scores for each factor represents the total psychological capital score. The higher the total score, the higher the level of psychological capital. The total Cronbach α coefficient of the questionnaire is 0.88, and the Cronbach coefficient of the total questionnaire in this survey sample is 0.876.

#### Children’s Strengths and Difficulties Questionnaire

The Children’s Strengths and Difficulties Questionnaire (student version; Goodman, 1997) was used to measure the children’s emotions and behaviors. The Strengths and Difficulties Questionnaire contains 25 items, categorized into five factors: emotional symptoms, conduct disorder, hyperactivity and impulsivity (aprosexia), peer interaction problems, and prosocial behavior. The sum of the former four factors represents the total difficulty score; the higher the total score, the greater the difficulty. For prosocial behavior, which is considered a positive quality, the higher the score, the greater the child’s capacity in this regard. The total Cronbach α coefficient of the questionnaire is 0.784, and the Cronbach coefficient of the questionnaire in this survey sample is 0.778.

### Test Procedure

This survey was conducted by project team members, with assistance from the head teachers from the target schools. Before the survey commenced, the head teachers explained the purpose of the survey to the subjects and their parents and obtained their consent. When the test was given to the elementary school students, the experimenter read the questions one-by-one and the subjects entered each answer immediately after the corresponding question had been read. The junior middle school students completed the questionnaire after receiving instruction from the experimenter. The questionnaires were completed and collected within approximately 30 min. This study was supported by the Child and Adolescent Health Promotion Research Center of Hunan Province.

### Data Processing and Statistical Methods

SPSS23.0 and MPLUS7.4 were used for data analysis. First, to remove the risk of systematic error caused by self-reporting bias, the Harman single-factor test was used to test the common method bias of the data. Second, MPLUS7.4 was used to analyze the children’s latent profiles and to classify them. All samples were divided into one class baseline model and analyzed; then, classified data were gradually added to fit the model. The main adaptation test indicators were likelihood ratio tests, the Akaike information criterion (AIC), the Bayesian information criterion (BIC), and the sample-size adjusted BIC; for these indicators, smaller values represent better adaptation. Entropy was used to estimate the accuracy of classification. For entropy, values close to 1 are desired; entropy greater than 0.8 indicates that the classification accuracy rate exceeds 90%. In addition, the Lo-Mendell-Rubin adjusted likelihood ratio test (LMRT) was performed. A statistically significant *p*-value indicates that the K-CLASS model is significantly better than the K-1-CLASS model (Li et al., 2014). Third, the latent class of psychological capital was used as the grouping variable to perform one-way analysis of variance for each index of emotional behavior, and to further conduct the multiple comparisons. This allowed us to explore differences in emotion and behaviors among the different latent classes of psychological capital. Finally, gender and grade were analyzed through ordinal logistic regression in order to test the effect of each indicator.

## Results

### Common Method Bias

A one-way analysis of variance showed that the six factors’ eigenvalue was greater than 1, and the variance of the first factor was 26.518%, less than the 40% critical value. This indicated that the data in this study were relatively unaffected by the common method bias.

### Result of the Latent Profile Analysis

The 25 items in the Rural Left-behind Children’s Psychological Capital Questionnaire were set as measurement indicators, and classes 1–4 (C1–C4) were selected for potential profile analysis. The results are shown in Table 1. The LMRT coefficient was statistically significant (*p <* 0.05) when the model was classified into two and three latent classes, but it was not statistically significant (*p* > 0.05) when it was divided into four latent classes. Compared with two latent classes, the entropy coefficient was greater and the AIC and BIC coefficients were smaller when the model was divided into three latent classes. Consequently, we felt that the model with three latent classes could fully describe the left-behind children’s psychological capital, and we determined that this was the optimum model. The attribution probability matrix of each latent class is shown in Table 2. For each latent class, the average attribution probability for the subjects was 94, 94, and 95%, respectively. This shows that the model with three latent classes was more reliable.

**TABLE 1:** Latent profile analysis of the differing psychological capital among the participants.

**C**	**K**	**Log(L)**	**AIC**	**BIC**	**aBIC**	**Entropy**	**LMRT (p)**	**BLRT (p)**	**Class probability**
1	50	−24640.18	49380.37	49606.25	49447.50				
2	76	−23423.49	46998.98	47342.32	47101.02	0.87	0.00	0.00	0.45/0.55
3	102	−23097.88	46399.75	46860.56	46536.70	0.88	0.03	0.00	0.11/0.46/0.43
4	128	−22952.73	46161.47	46739.73	46333.31	0.85	0.09	0.00	0.20/0.09/0.29/0.42

*K indicates the number of freely estimated parameters. AIC, Akaike information criterion; BIC, Bayesian information criterion; aBIC, adjusted Bayesian information criterion; BLRT, bootstrap likelihood ratio test; LMRT, Lo-Mendell-Rubin adjusted likelihood ratio test.*

**TABLE 2:** Average attribution probability per subject for each potential class.

**Subject**	**C1 (%)**	**C2 (%)**	**C3 (%)**
**probability**			
C1	0.94	0.06	0.00
C2	0.02	0.94	0.04
C3	0.00	0.05	0.95

**FIGURE 1:**
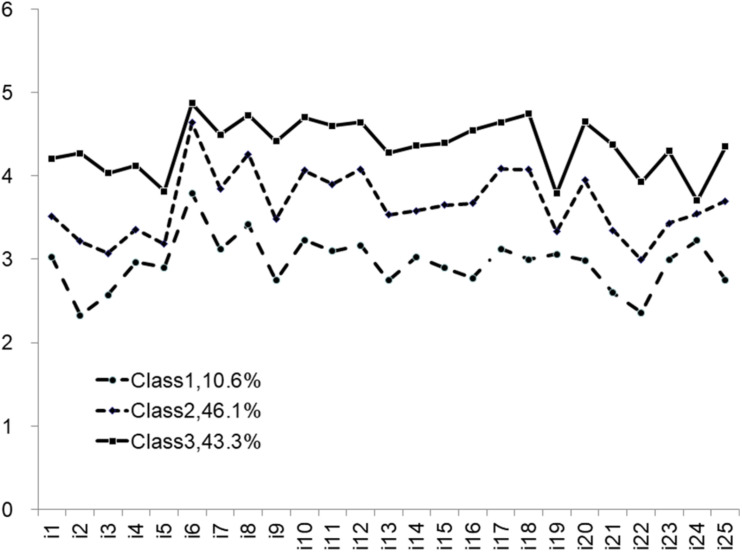
Mean scores of the three latent classes for each of the 25 items of the Rural Left-behind Children’s Psychological Capital Questionnaire.

Figure 1 shows that there were significant differences between the three latent classes in terms of the mean scores for each of the 25 items of the Rural Left-behind Children’s Psychological Capital Questionnaire. For C1, the mean score for each item was significantly lower than those of the other two classes, which indicated a lower level of psychological capital. Therefore, C1 was named “low psychological capital.” Seventy children were categorized into this class, accounting for 10.6% of all participants in the sample. The mean scores for C2 were higher than those for C1 but lower than those for C3; thus, C2 was named “medium psychological capital.” Three hundred and eleven children were included in this class, accounting for 46.1% of all subjects. Finally, the mean scores for C3 were significantly higher than those of both C1 and C2, and C3 was consequently named “high psychological capital.” This class contained 296 left-behind children, accounting for 43.3% of all subjects.

### Comparison of the Different Latent Classes of Psychological Capital in Terms of Their Respective Characteristics and Differences Regarding Emotional and Behavioral Problems

A one-way analysis of variance was performed to compare, among children with different latent classes of psychological capital, emotional and behavioral indicators, and the results are shown in Table 3. This showed that there were significant differences between the different subtypes of psychological capital in terms of emotional symptoms, conduct disorder, hyperactivity and impulsivity, peer-interaction problems, total difficulty score, and prosocial behavior (*p* < 0.001).

**TABLE 3:** Comparison among the different latent classes of psychological capital in terms of characteristics and differences regarding emotional and behavioral problems.

**Item**	**Emotional symptoms**	**Conduct disorder**	**Hyperactivity and impulsivity**	**Peer interaction problems**	**Total difficulty score**	**Prosocial behavior**
Condition	3.11 ± 2.32	2.88 ± 1.83	3.96 ± 1.98	3.79 ± 1.58	13.74 ± 5.27	6.32 ± 2.29
High (a)	4.04 ± 2.16	3.27 ± 1.79	5.27 ± 1.97	4.19 ± 1.65	16.77 ± 4.66	4.67 ± 1.75
Medium (b)	3.22 ± 2.30	3.04 ± 1.68	4.34 ± 1.78	3.86 ± 1.57	14.46 ± 4.88	5.83 ± 2.13
Low (c)	2.78 ± 2.33	2.62 ± 1.96	3.24 ± 1.92	3.62 ± 1.56	12.26 ± 5.36	7.23 ± 2.19
*F*-value	9.17***	5.84**	45.95***	4.23*	28.21***	56.56***
LSD	a > b > c	a ≈ b > c	a > b > c	a > b ≈ c	a > b > c	a < b < c

*p < 0.05, **p < 0.01, ***p < 0.001.

### Gender and Age Effects of the Latent Classes

Based on the results of the above analysis of the latent classes of psychological capital, we further explored the effects of gender and grade. The results of the three latent classes were used as dependent variables, and the gender and grade indicators were used as independent variables. The odds-ratio coefficient was obtained by using high psychological capital as the benchmark. The odds-ratio value reflected the effects of gender, grade, and latent class. The results are shown in Table 4. Compared to the children with low psychological capital, the proportion of elementary school children with high psychological capital was greater, but there was no gender-related difference present.

**TABLE 4:** Ordinal logistic regression of factors influencing the latent classes of psychological capital.

	**Medium**			**High**		
	**OR**	***p***	**95%CI**	**OR**	***p***	**95%CI**
Gender (F)	1.31	0.31	0.78–2.21	1.31	0.32	0.77–2.22
Grade (1–6)	1.47	0.16	0.86–2.49	2.29	0.002	1.34–3.90

*The dependent variables are based on the latent class of low psychological capital, gender (male = 1, female = 0), and grade (junior middle = 1, primary = 0).*

*CI, confidence interval; OR, odds ratio.*

## Discussion

In this study, differences in positive psychological resources and subtypes of psychological capital were found among left-behind children in schools in rural areas of China. Analysis of existing research indicates that this is the first study to explore the latent classes of psychological capital among left-behind children. Consistent with other symptom studies, we found several different latent classes of left-behind children in this sample, which provides a basis for clarifying the differing psychological capital levels that exist among left-behind children. Specifically, LPA results supported a model with three classes, comprising high, medium, and low psychological capital, respectively. These findings not only support the classification of left-behind children’s psychological capital, but also show the differences in psychological capital levels among different latent classes.

In general, the profile lines of the three categories in this research model show a relatively consistent trend. However, a careful comparison of the item content reveals that in the “negative” category, Items 2 and 9 (Items 6 and 17, respectively, in the original scale) (reverse-scored) is particularly low. These two items are “I can endure all kinds of difficulties” and “No matter what I do, I can’t let my parents down.” Item 19 (also Item 19 in the original scale), “I can’t see hope for my own future,” has a higher conditional probability. This shows that among the left-behind children with low psychological capital, positive psychological qualities, such as mental toughness, understanding, and sense of hope, are low.

Notably, in these three categories, Item 6 (Item 2 in the original scale, reverse-scored), “I often want to repay my parents and other elders in the future” showed higher conditional probability, while Item 22 (Item 10 in the original scale, reverse-scored), “I feel very optimistic, and there is almost no time to be depressed,” showed low conditional probability. In terms of optimism, the conclusions are consistent, indicating that left-behind children have low positive emotions and need to be taken seriously.

In this study, over 43.3% of the children showed a high level of psychological capital, and were defined as “children with high psychological capital.” Coincidentally, in the research on the excellent qualities of children and adolescents, the researchers found that about 40% of children and adolescents performed well in various excellent qualities, which is very close to the conclusion of this study (Liu et al., 2016). These children had relatively higher levels of self-reliance and perseverance, sensibility and appreciation, optimism and cheerfulness, tolerance and kindness, and self-confidence and aggressiveness, which indicated that they have more positive psychological resources, more social support, greater adaptability, greater optimism about life, and greater hope for the future. Meanwhile, 46.1% of the left-behind children showed a good level of psychological capital, and were defined as “children with medium psychological capital.” Such children show good adaptability; however, compared with the children with high psychological capital, they may receive less social support, are less optimistic about life, and have less hope for the future. Thus, they require attention and appropriate intervention. The percentage of the sample accounted for by these two kinds of children indicates that over 80% of the children had above medium-level psychological capital. Ecological systems theory highlights that a change in individual environment has an important influence on children’s development; such change can be instigated by parents moving to a city or parent-child separation at an early stage. During this transitional period, children will experience challenges, self-challenges, and positive adaptation. In this process, individual psychological adaptation may be influenced by the interaction of one’s individual characteristics with the different systems he/she is consequently exposed to, such as family members and peers (Bronfenbrenner, 1979). In addition, the previous theory suggests that psychological emotional disorders can be caused by emotional attachment. Left-behind children whose parents have been away for many years may suffer more emotional neglect and emotional deprivation than their peers. During adolescence, patterns of interpersonal interactions adjust continuously with the emergence of new attachment objects (DeLuca et al., 2019). As left-behind children adapt to the lack of parental attachment, their interpersonal interactions can gradually shift from a parental focus to a peer focus (Graham and Jordan, 2011). In addition, as a result of the boarding system that is applied in rural Chinese schools and attention from the government, positive peer interaction and strong peer attachment are found among left-behind children who receive more social support. This arrangement results in constant improvement of positive qualities such as mental toughness and self-efficacy, which promote psychological capital and represent protective factors for mental health. Importantly, 10.6% of the left-behind children in our study showed lower levels of psychological capital than the other two classes, and these were defined as “children with low psychological capital.” This study speculated that such children are less adaptable to society, are pessimistic regarding school life, are diffident, and have less hope for the future.

The LPA results suggest that there are intergroup differences regarding left-behind children’s psychological capital levels. Most left-behind children have good psychological development because of the attention and support they receive from society; however, 10% are at risk of poor mental development. Left-behind children with medium psychological capital must receive attention and assistance to help them develop high psychological capital. Meanwhile, for left-behind children with low psychological capital, efforts must be made to identify and address their problems. On one hand, their psychological capital must be protected and deterioration of their mental health must be avoided. On the other hand, it is also necessary to take actions to help them build self-confidence and improve self-efficacy and psychological resilience, which can improve their mental health.

This study also found that, for left-behind children in China, there are significant differences among the different latent classes of psychological capital regarding emotional and behavioral indicators. Children with low psychological capital have more emotional symptoms, hyperactivity and impulsivity, and peer-interaction problems, tend to have conduct disorder, and are less prosocial. Previous studies have also confirmed this view (Xu, 2016). The Conservation of Resources Theory (Hobfoll, 2011; Wu et al., 2012) argues that, to manage the negative effects of various stressors, individuals must mobilize their positive resources. However, resource-poor people may lack the necessary reserves to manage stress and, by exhausting their reserves, accelerate their loss of resources and enter loss spirals. Left-behind children with low psychological capital often have limited positive psychological resources and cannot effectively address the risks and crises they encounter; consequently, they develop relatively more emotional and behavioral problems. In contrast, individuals with certain good resources (such as optimism and confidence) have the ability to access other resources, and their good resources will also generate additional resource increments (Przybylski and Weinstein, 2019), meaning they have sufficient psychological capital to address various risks and crises. Thus, left-behind children with high psychological capital are more likely to have fewer emotional and behavioral problems. This finding supports the theory that low psychological capital can cause emotional and behavioral problems in left-behind children, underlining the applicability of the Psychological Capital Intervention theory (Russo and Stoykova, 2015).

According to the results of this study, interventions to increase psychological capital can be carried out using three different approaches. The first is to start with emotions and guide left-behind children to master some necessary emotional regulation and management methods. Studies have found that writing, exercise, and relaxation are three effective ways to express emotions, which can help individuals reduce negative emotions and learn to face the future positively and optimistically. The second is to start with daily behaviors and encourage positive behaviors through an effective reward-and-punishment system, to decrease negative behaviors and help these children become more confident, enterprising, self-reliant, and tenacious. The third is to help left-behind children establish good interpersonal relationships, increase prosocial behaviors, improve relationships with peers, and obtain more social support, thereby guiding them to become more tolerant, friendly, and grateful.

Finally, this study also explored the impact of gender and grade on the different subtypes of left-behind children’s psychological capital, and found that the proportion of primary school students in the high psychological capital of the left-behind children was greater. A possible reason for this result is that left-behind junior high school students are entering adolescence, a period when individual physiological changes, interpersonal communication, academic stress, social expectations, and other psychosocial factors can bring various forms of stress. These kinds of stress and adverse cognitive factors (Yuan et al., 2014) cause left-behind junior high school students who have limited positive psychological capital to become unable to address their problems, resulting in low psychological capital. Notably, many left-behind children in rural areas of China, after graduating from junior high school, become migrant workers instead of pursuing higher education. Thus, more attention should be paid to this issue.

Through analysis of the latent classes of left-behind children’s psychological capital, this study aimed to prove that there are intergroup differences in left-behind children’s psychological capital. The results of this research can provide a basis for classifying left-behind children and providing them with appropriate education, and can also contribute to the development of interventions that can cultivate children’s positive psychological qualities and improve their psychological capital. Of course, there are still some limitations in this study. The samples in this study were limited to Hunan Province, and samples from other regions were not included. In addition, the potential profile analysis method itself was more susceptible to the influence of samples, so it needs to be further verified in other samples. At the same time, according to the theory of human-environment interaction, we can further explore the joint influence of individual factors and environmental factors on psychological capital.

## Data Availability Statement

The raw data supporting the conclusions of this article will be made available by the authors, without undue reservation.

## Ethics Statement

The studies involving human participants were reviewed and approved by the Academic Committee of the College of Education of Hunan Agricultural University. Written informed consent to participate in this study was provided by the participants’ legal guardian/next of kin.

## Author Contributions

CZ was mainly responsible for the overall conception and design of this study. XY wrote the manuscript and carried out the statistical analysis. LZ and ZL were responsible for the questionnaire survey and sorting out. All authors contributed to the article and approved the submitted version.

## Conflict of Interest

The authors declare that the research was conducted in the absence of any commercial or financial relationships that could be construed as a potential conflict of interest.
